# A novel deletion in *FLOWERING LOCUS T* modulates flowering time in winter oilseed rape

**DOI:** 10.1007/s00122-021-03768-4

**Published:** 2021-01-20

**Authors:** Paul Vollrath, Harmeet S. Chawla, Sarah V. Schiessl, Iulian Gabur, HueyTyng Lee, Rod J. Snowdon, Christian Obermeier

**Affiliations:** Department of Plant Breeding, Justus Liebig University, Giessen, Germany

## Abstract

**Key message:**

A novel structural variant was discovered in the FLOWERING LOCUS T orthologue BnaFT.A02 by long-read sequencing. Nested association mapping in an elite winter oilseed rape population revealed that this 288 bp deletion associates with early flowering, putatively by modification of binding-sites for important flowering regulation genes.

**Abstract:**

Perfect timing of flowering is crucial for optimal pollination and high seed yield. Extensive previous studies of flowering behavior in *Brassica napus* (canola, rapeseed) identified mutations in key flowering regulators which differentiate winter, semi-winter and spring ecotypes. However, because these are generally fixed in locally adapted genotypes, they have only limited relevance for fine adjustment of flowering time in elite cultivar gene pools. In crosses between ecotypes, the ecotype-specific major-effect mutations mask minor-effect loci of interest for breeding. Here, we investigated flowering time in a multiparental mapping population derived from seven elite winter oilseed rape cultivars which are fixed for major-effect mutations separating winter-type rapeseed from other ecotypes. Association mapping revealed eight genomic regions on chromosomes A02, C02 and C03 associating with fine modulation of flowering time. Long-read genomic resequencing of the seven parental lines identified seven structural variants coinciding with candidate genes for flowering time within chromosome regions associated with flowering time. Segregation patterns for these variants in the elite multiparental population and a diversity set of winter types using locus-specific assays revealed significant associations with flowering time for three deletions on chromosome A02. One of these was a previously undescribed 288 bp deletion within the second intron of *FLOWERING LOCUS T* on chromosome A02, emphasizing the advantage of long-read sequencing for detection of structural variants in this size range. Detailed analysis revealed the impact of this specific deletion on flowering-time modulation under extreme environments and varying day lengths in elite, winter-type oilseed rape.

**Supplementary information:**

The online version contains supplementary material available at (10.1007/s00122-021-03768-4).

## Introduction

Structural genome variation (SV) is widespread in crop genomes and frequently associated with important traits (Gabur et al. 2019). Recent improvements in genome sequencing enable detection of SV larger than 30 base pairs (Chawla et al. 2020). Different types of SV, including deletions, insertions, duplications, translocation and inversions (Feuk et al. 2006), can have a substantial effect on gene expression and accordingly on the phenotypes of plants (Alonge et al. 2020; Saxena et al. 2014; Wang et al. 2016). Examples include determination of different agronomic traits such as flowering time (Díaz et al. 2012; Schiessl et al. 2017b; Song et al. 2020), disease resistance (Gabur et al. 2018; Hurgobin et al. 2018), heading date (Nishida et al. 2013), seed weight (Song et al. 2020) and biotic stress response (McHale et al. 2012). Due to their cumulative size, more nucleotides tend to be affected by SV than by SNP variants on a genome-wide scale (De Coster et al. 2019).

*Brassica napus* (2*n* = 4*x* = 38, AACC) is an allotetraploid interspecific hybrid between *Brassica rapa* (2*n* = 2*x* = 20, AA) and *Brassica oleracea* (2*n* = 2*x* = 18, CC) (Chalhoub et al. 2014; Parkin et al. 1995; U 1935). Due to the high similarity between the A and the C subgenomes, homoeologous exchanges occur very frequently (Chalhoub et al. 2014; Hurgobin et al. 2018; Stein et al. 2017; Szadkowski et al. 2010). Many studies to date have focused on SV in resynthesized oilseed rape, which is known to be affected by numerous and large variation. SV has been visualized using genetic mapping (Song et al. 1995; Gabur et al. 2020), molecular cytogenetics (Xiong et al. 2011), optical mapping (Gabur et al. 2020) and genome-wide short-read sequencing (Chalhoub et al. 2014; Samans et al. 2017), but all of these methods are only capable of accurately detecting large-scale SV (> 100,000 bp; Samans et al. 2017). However, using long-read sequencing, we recently demonstrated that small-scale SV events (30–10,000 bp) are also unexpectedly widespread in natural *B. napus* (Chawla et al. 2020), with considerable implications for gene function and diversity. Sequence-capture experiments demonstrated a major impact of SV on flowering-time regulatory pathway genes (Schiessl et al. 2017a, b), which play a key role in crop adaptation and yield performance. This study aims to capture the relevance of genome-wide SV in elite European winter oilseed rape and investigate the specific impact of SV on flowering time in a narrow breeding gene pool.

Facing climate change and the trend toward increasingly warmer winters in Europe, in conjunction with an earlier onset of vegetative growth after winter, flowering time is a crucial factor for plant development, seed formation and ultimately yield production. However, flowering time is determined by many genes, which complicates its examination (Blümel et al. 2015). Previous studies on flowering time in *B. napus* focused primarily on identification of major genetic factors responsible for differentiation of winter, semi-winter and spring morphotypes (Long et al. 2007; Raman et al. 2019; Schiessl et al. 2017b, 2019; Song et al. 2020; Wu et al. 2019; Xu et al. 2016). Due to the strong effects of major mutations in key flowering-time regulators, which are jointly responsible for ecogeographical differentiation, minor effects of allelic differentiation tend to be obscured in such studies. For breeding, however, where fine-tuning of flowering within ecotypes is critical, dissection of minor-effect loci in elite breeding gene pools can be more relevant than major-effect loci which disturb local adaptation. Therefore, in this study we deliberately carried out an analysis of flowering-time variation in elite winter cultivars, aiming to find novel genomic regions for fine adjustment of flowering modulation. We first investigated SV events associated with flowering-time variation in a double haploid (DH) multiparental association mapping population derived from crossing six inbred lines from elite commercial cultivars to a common parent (also a modern elite cultivar). Single-locus assays of interesting SV were further assessed for their impact on flowering time in a winter-type *B. napus* diversity set. The results revealed associations of SV with the onset of flowering in a number of flowering-time genes within QTL regions in elite oilseed rape. The findings can help to breed for adapted oilseed rape cultivars that fulfill current and future requirements in the course of climatic changes.

## Material and methods

### Plant material

A multiparental population comprising 354 DH lines was used in this study (Supplementary Table S1 and Supplementary Fig. 1). The panel consists of six subpopulations derived by crossing six elite founder lines (‘Adriana’, ‘Alpaga’, ‘DK Cabernet’, ‘Galileo’, ‘King 10′ and the DH line ‘JN’) to the common elite parent ‘Lorenz’. DH families from the crosses King 10 × Lorenz, Adriana × Lorenz, and JN × Lorenz were produced by NPZ Innovation GmbH (Holtsee, Germany). DH families from the crosses Lorenz × Alpaga and Lorenz × DK Cabernet were produced by Syngenta Seeds GmbH (Bad Salzuflen, Germany), while the family from the cross Lorenz × Galileo was produced by KWS SAAT SE (Einbeck, Germany). All six subpopulations comprised 60 DH lines except for the cross Lorenz × Galileo, which comprised 54 DH lines. In addition, we also investigated a set of 140 genetically diverse inbred lines of winter-type oilseed rape selected from the ERANET-ASSYST *B. napus* diversity set described in Körber et al. (2012) (Supplementary Table S2).

### Field trials and phenotyping data

Field trials were conducted in an alpha lattice design with two replications at five independent locations across Germany in the 2017/2018 and 2018/2019 growing seasons. Plants were grown in plots of 6 m^2^ (4 × 1.5 m) with a sowing density of 45 seeds per m^2^. In 2017/18, the trials were located in Hadmersleben (Syngenta Seeds GmbH) and Nienstädt (Monsanto Agrar Deutschland GmbH), while in 2018/19 trials were carried out in Hovedissen (W. von Borries-Eckendorf GmbH & Co. KG), Leutewitz (Deutsche Saatveredelung AG) and Soerup (Bayer CropScience AG). In addition, we accessed flowering-time data from Schiessl et al. (2015) for 140 winter-type lines of the ERANET-ASSYST diversity set assessed over a total of 13 environments from 2010 to 2013, including flowering-time data from Giessen (Germany) in 2011 and 2012, Gross Gerau (Germany) in 2010, 2011 and 2012, Beibei (China) and Temuco (Chile) in 2011 and 2012, and Rauischholzhausen (Germany) in 2010, 2011, 2012 and 2013. We further assessed the diversity set for flowering time in a randomized complete block design with a plot size of 12.5 m^2^ (10 × 1.25 m) in one replicate in Rauischholzhausen (Germany) in 2018. Start of flowering was defined as BBCH 61 (10% of flowers on main raceme open) (Lancashire et al. 1991) and is reported in all trials as the number of days after sowing.

### SNP genotyping and linkage analysis

The entire population including the seven parents was genotyped using the *Brassica* 60 k Illumina Infinium™ SNP array. By allowing zero mismatches and gaps, a stringent alignment using BLASTN (Altschul et al. 1990) was conducted and we were able to anchor 27,832 SNP uniquely to a single position of the publicly available *B. napus* genome assembly Darmor-*bzh* v4.1 (Chalhoub et al. 2014). Failed and nonspecific SNP were excluded from further analysis. Using the software Haploview, we defined linkage disequilibrium (LD) blocks based on their confidence intervals according to Gabriel et al. (2002).

### Genome-wide association studies (GWAS)

The R package GenABEL (Aulchenko et al. 2007) was used to conduct a genome-wide association study (GWAS). Markers with more than 10% missing data or a minor allele frequency lower than 5% were excluded, as were genotypes with more than 10% missing data. The kinship and principal component analysis were included in the model to adjust for population stratification. We applied a false discovery rate (FDR) of ≤ 0.1 (Benjamini and Hochberg 1995) to call a marker–trait association significant. In addition, to reduce the type II error rate, a threshold of − log_10_ (*p* value) ≥ 3.0 for marker–trait associations was defined to consider a marker as a putative SNP. Phenotypic variance (*R*^2^) was estimated using the formula ${R^2}\; = \;\frac{\chi }{{n - 2 + \chi }}$. As shown by Gabur et al. (2018), calling of single nucleotide absence polymorphisms (SNaP) by segregation patterns can provide additional markers and reveal previously undetected QTL in *B. napus*. Using this procedure, we called SNaP markers for loci that failed the threshold of < 10% failed calls but showed the expected 1:1 segregation for presence–absence polymorphisms in the respective subfamilies.

### Whole genome sequencing and variant calling

Long-read sequencing on the MinION device from Oxford Nanopore Technologies (ONT) (Oxford, UK) was used to sequence genomic DNA from each parental line to a genome coverage of at least 20 ×. For this purpose, high molecular weight (HMW) DNA isolation was performed using a protocol modified from Mayjonade et al. (2016), as described in Chawla et al. (2020). In order to achieve a longer average read length, we conducted a size selection step using the Circulomics Short Read Eliminator Kit (https://www.circulomics.com/sre) prior to the sequencing library preparation. The kit consists of a buffer that separates small DNA fragments by centrifugation. The size-selected HMW-DNA was subsequently used to prepare the sequencing library. We utilized the SQK-LSK109 sequencing kit provided by Oxford Nanopore Technologies using their recommended protocol. Library preparation consisted of the following two steps: end repair and adapter ligation, each followed by a cleaning step with magnetic beads. Finally, a library of 500–1000 ng of HMW-DNA was transferred into the MinION flow cell for sequencing. MinION raw signal data were processed using the base-caller Guppy version 3.2.1 (https://community.nanoporetech.com/downloads/guppy/release_notes). Read quality was evaluated with the tool MinIONQC (Lanfear et al. 2019), and raw reads with a minimum Q score of 7 were aligned to the *B. napus* reference genome Darmor-*bzh* v4.1 (Chalhoub et al. 2014) using the NGMLR long-read mapper version 0.2.7 (Sedlazeck et al. 2018). Subsequently, the alignment file in SAM format was converted to a sorted BAM file using SAMtools version 1.9 (Li et al. 2009) and genome-wide SV was called using Sniffles version 1.0.10 (Sedlazeck et al. 2018). Settings and subsequent filtering of SV followed Chawla et al. (2020) with a focus only on deletions and insertions. Bedtools version 2.29.2 was used to detect SV overlapping with genes (Quinlan et al. 2010). Using the Integrative Genomics Viewer version 2.6.1, we produced coverage plots and illustrated SV and genes (Robinson et al. 2011). The segregation ratio of SV was tested conducting a χ^2^ test. The online database JASPAR was used to screen for transcription factor binding sites (http://jaspar.genereg.net/) (Khan et al. 2018).

### SV validation

To validate interesting SV events detected by the variant calling, we used a standard PCR approach using primers flanking the deletions/insertions. Primer design was performed using Primer3Plus (Untergasser et al. 2007) and BLAT (Kent 2002) via the *Brassica napus* Genome Browser (http://www.genoscope.cns.fr/brassicanapus). All primer sequences can be found in Supplementary Table S3. Sanger sequencing of PCR products was conducted to confirm unique primer binding. Besides PCR, we confirmed the 288 bp deletion in *FLOWERING LOCUS T* on chromosome A02 (*BnaFT.A02*; *BnaA02g12130D*) using 100 bp single-end Illumina short read sequencing data from 140 inbred winter-type lines of the diversity set (Schiessl et al. 2017b). Short reads from genotypes carrying the deletion fail to map accurately to *BnaFT.A02* in the Darmor-*bzh* version v4.1 reference genome due to the deleted 288 bp and 92 unresolved nucleotides (Ns) within the reference. Therefore, we modified the reference by adding a corrected version of *BnaFT.A02* using long read data from cultivar Express 617 (Lee et al. 2020), once with and once without the 288 bp sequence. Alignment, removal of duplicates, sorting, indexing and normalized mean coverage (NMC) calling for the 288 bp position were performed according to Schiessl et al. (2017c). An NMC value below 0.5 indicated association with a confirmed deletion.

## Results

### GWAS for identification of flowering-time QTL

**Fig. 1:**
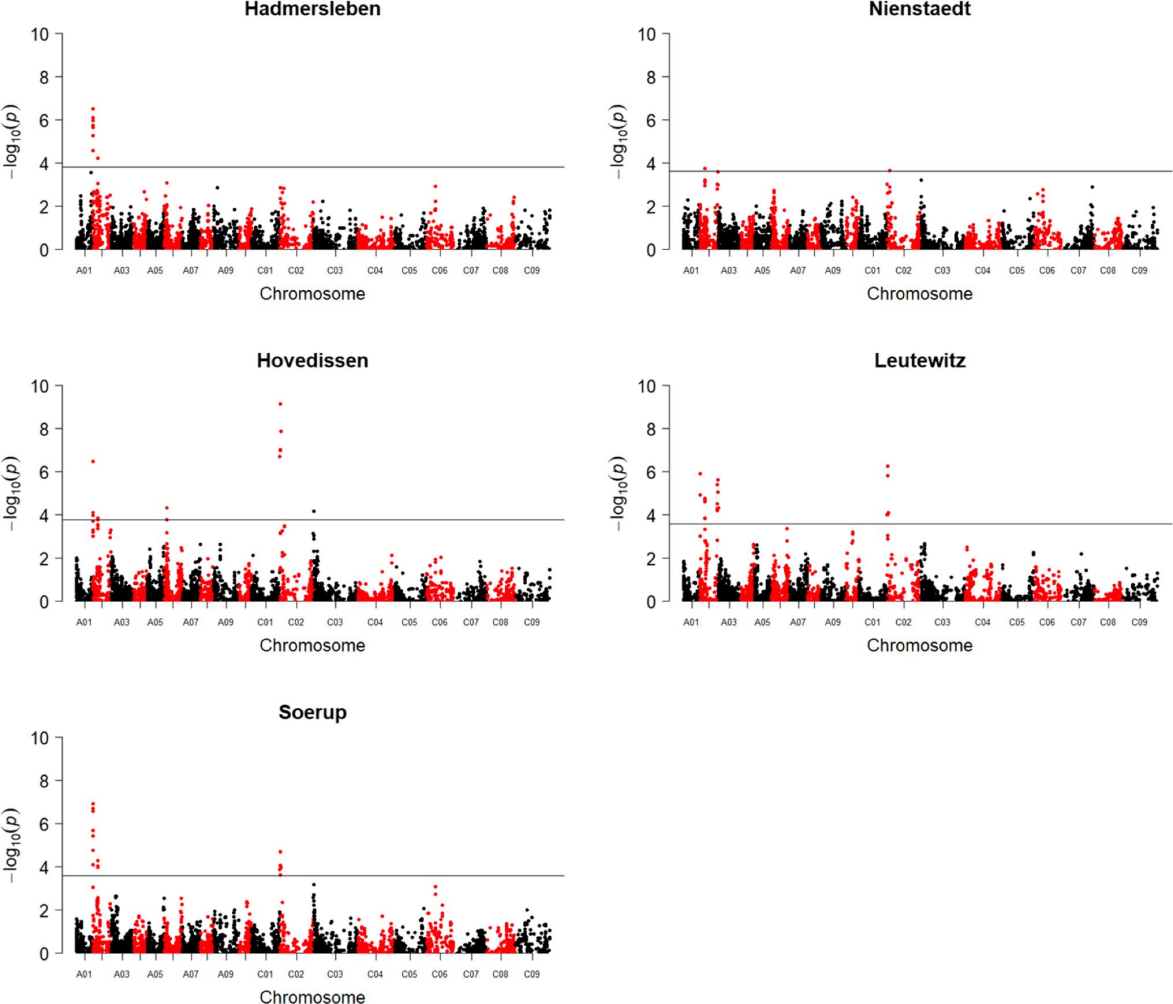
Manhattan plots showing marker–trait associations for flowering time (days after sowing) in the multiparental population in five independent environments across Germany (*n* = 352). The solid line represents the threshold for significant SNP markers (FDR ≤ 0.1)

A total of 13,746 high-quality polymorphic SNP markers were identified in the mapping population. Additionally, 578 SNaP markers were called by analyzing the segregation patterns of raw array data in the subpopulations, as described in Gabur et al. (2018). Using all 14,324 markers, eight genomic regions associated with flowering time in winter oilseed rape were detected in at least two of five field environments (Fig. 1 and Supplementary Table S4). Four of the detected QTL were located on chromosome A02, in LD blocks with sizes of 352 kb, 498 kb, 451 kb and 383 kb. Three QTL were identified on chromosome C02 in LD blocks of 331 kb, 860 kb and 360 kb, respectively, while a 914 kb QTL interval was detected at the distal end of chromosome C03. Broad sense heritability (Falconer and Mackay 1996) for the onset of flowering was high (*h*^2^ = 0.83).

### Genome-wide structural variant detection in seven elite parental lines using long-read sequencing

**Fig. 2:**
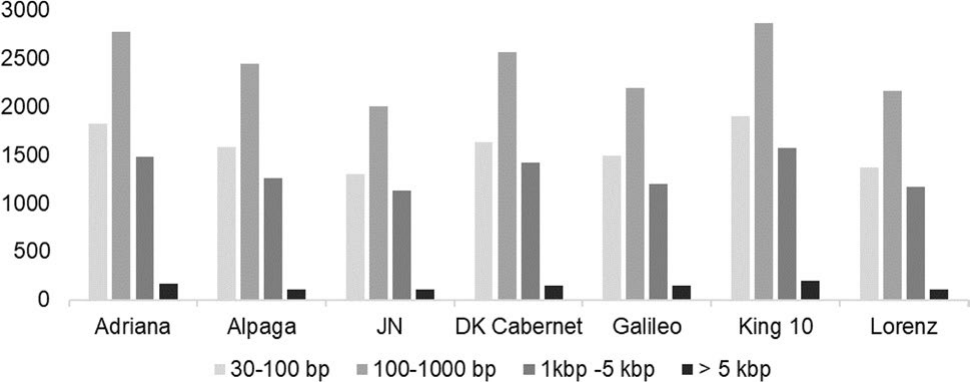
Total numbers of polymorphic SV detected in seven parental lines by comparison to the *B. napus* reference Darmor-*bzh* v4.1 grouped in four different size classes

A total of 250 Gb of raw sequencing data was generated from 29 MinION flowcells, with an average yield of 35.7 Gb per genotype. A genome coverage of 23 × to 43 × was achieved for the seven parents of the mapping population. The first 20 flowcell runs delivered average N50 values of 21,967 bp. After using the Circulomics Short Read Eliminator Kit for removing short reads from the library in the following nine sequencing runs, the N50 increased to an average of 30,526 bp, so that the final N50 of the cumulative data from all flowcells ranged from 20,418 to 29,260 bp for all seven genotypes. A summary of sequencing results including read numbers, read lengths and the quality of the reads can be found in Supplementary Table S5. Mapping rates using the Darmor-*bzh* v4.1 reference genome ranged from 63 to 72% for the seven parental lines (Supplementary Table S6). Variant calling revealed a total of 50,762 unique SV across the seven parental lines compared to Darmor-*bzh*, of which 13,374 were polymorphic across the seven parental lines. Conversely, 37,388 SV calls were consistent for all seven parental lines and therefore monomorphic in the population, but different to the Darmor-*bzh* reference; these calls likely include assembly errors of Darmor-*bzh*. Of the polymorphic SV events we detected, 6,990 were deletions and 6,384 were insertions, with sizes ranging from 30 bp to 26 kb. The majority (47.5%) had a size between 100 and 1,000 bp (Fig. 2). Median sizes for deletions and insertions were 429 bp and 263 bp, respectively. All detected polymorphic deletions and insertions were included in subsequent analyses.

### Genes affected by SV within flowering-time QTL

To explore the potential impact of the genome-wide SV on protein coding genes, we firstly compared the positions of the detected SV with the gene regions for 101,040 genes annotated in the Darmor-*bzh* reference genome (Chalhoub et al. 2014). In total, 4,840 (4.8%) of all annotated genes in the genome coincided with polymorphic SV events in the parental lines of the mapping population. A similar frequency of genes coinciding with SV events was observed within the eight flowering-time QTL (6.7%, 52 of 771). To investigate whether flowering time might be affected by intragenic SV, we analyzed the genes within QTL regions in more detail. In total, we found 771 annotated genes contained within the eight QTL regions, spanning 1.684 Mb on chromosome A02, 1.551 Mb on chromosome C02 and 914 kb on chromosome C03. By filtering gene ontology (GO) terms of the *B. napus* gene annotations on these 771 genes for terms that can be linked to flowering time, we identified 71 genes annotated to be involved in flowering-time regulation, among them copies of the vernalization gene *FLOWERING LOCUS C* (*FLC*) and the circadian rhythm gene *LATE ELONGATED HYPOCOTYL* (*LHY*) (Table 1 and Supplementary Table S7). Six out of these 71 genes exhibit SV. Of these six genes, five were detected in the QTL regions on chromosome A02 and one in the QTL region on chromosome C02. The affected gene on chromosome C02 is not homoeologous to any of the five flowering-related genes on chromosome A02, and the QTL regions on A02 and C02 do not represent homoeologous regions based on comparison of harbored genes as defined by Chalhoub et al. (2014). By analyzing regions up to 10 kb upstream of the start codons of the flowering-time genes, we found a large 1,361 bp deletion in the parental line JN in the promoter region of *BnaFT.A02* (*BnaA02g12130D*) (Table 1). In three of the seven parental lines, the same gene also displayed a 288 bp deletion in the second intron.

**Table 1:** Flowering-time candidate genes and coinciding SV identified between 7 elite parental lines within QTL for flowering time analyzed in a *B. napus* multiparental elite population (*n* = 354)

QTL name	Size of LD block in kb	Start–end position	Number of genes/flowering-time genes in Darmor-*bzh*	Number of genes/flowering-time genes with structural variants*	Gene IDs	Blast2GO annotation of gene	Name: size and type of SV (relative to Darmor-*bzh*)	PCR assay name: product size polymorphism (see Fig. 3)
A02_1	352	12,297–364,239	92/10 (10.9%)	14 / 2 (14.2%)	*BnaA02g00160D*; *BnaA02g00910D*	Pollen development; regulation of flower development	SV1: 373 bp deletion; SV2: 64 bp deletion	P133: 1130 bp/757 bp; P255: 419 bp/362 bp
A02_2	498	6,311,854–6,810,319	59/8 (13.6%)	6/1 (16.7%)	Promoter of *BnaFT.A02*; *BnaA02g12130D* (*BnaFT.A02*)	-; photoperiodism, flowering, positive regulation of flower development	SV3: 1,361 bp deletion; SV4: 288 bp deletion	P311: 1924 bp/563 bp; P416: 797 bp/519 bp
A02_3	451	22,569,685–23,021,163	73/3 (4.1%)	2/0 (0%)	–	–	–	–
A02_4	383	24,112,932–24,495,807	72/11 (15.3%)	5 / 2 (40%)	*BnaA02g33650D*; *BnaA02g33940D*	Circadian rhythm; entrainment of circadian clock	SV5: 1,313 bp deletion; SV6: 35 bp deletion	P513: 819 bp/no product; P632: 667 bp/632 bp
C02_1	331	512,169–842,796	70/0 (0%)	2/0 (0%)	–	–	–	–
C02_2	860	1,028,042–1,888,045	153/11 (7.2%)	2/1 (50%)	*BnaC02g03640D*	Regulation of flower development	SV7: 38 bp insertion	P732: 262 bp/300 bp
C02_3	360	3,168,965–3,528,564	62/5 (8.1%)	10/0 (0%)	–	–	–	–
C03_1	914	2,434–915,986	190/23(12.1%)	11/0 (0%)	–	–	–	–
Sum	4,149	-	771/71 (9.2%)	52/6 (11.5%)	–	–	–	–

Flowering-time genes were defined according to Schiessl et al. (2015) as genes associated by Blast2GO with at least one of the following gene ontology terms: flower, vernalization, photoperiod, circadian, floral, vegetative to reproductive, vegetative phase change, pollen carpel, sepal, petal

### Association of intragenic deletions with flowering time in the multiparental population

**Fig. 3:**
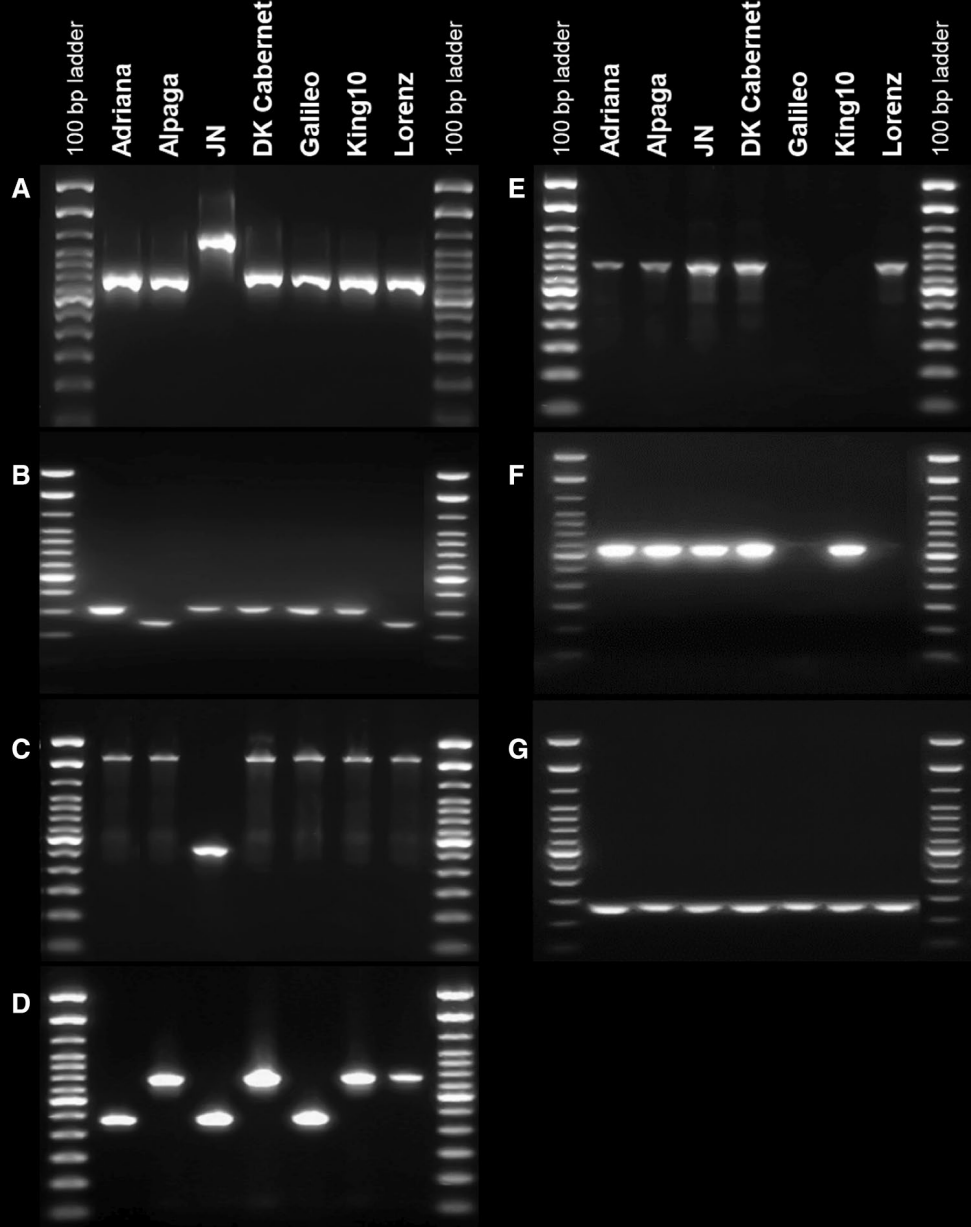
Size polymorphism within six flowering-time genes from three QTL on A02 and one QTL on C02 detected by PCR in seven parents of the *B. napus* multiparental population with the common parent Lorenz. **a** SV1: 373 bp deletion, **b** SV2: 64 bp deletion, **c** SV3: 1,361 bp deletion, **d** SV4: 288 bp deletion, **e** SV5: 1,313 bp deletion, **f** SV6: 35 bp deletion, **g** SV7: 38 bp insertion (also see Tables 1 and 2 for more details)

To evaluate trait associations with deletions in flowering-time genes, we developed specific PCR assays for all seven SV events in the six QTL-linked flowering-time candidate genes and used these to analyze the segregation of size polymorphisms identified in the parental lines in relevant subpopulations (Fig. 3, Supplementary Table S3). The seven PCR assays were first validated in the seven parental lines of the multiparental population. In 47 of these 49 PCR assays, the alleles called by ONT data from the parental lines were reconfirmed, whereas a 38 bp insertion, designated SV7, could not be reconfirmed in parental lines King 10 and Galileo and was excluded from further analyses. The PCR assays were then applied to the respective subpopulations which showed the corresponding polymorphism and tested for the expected 1:1 segregation. In most cases, the observed segregation in the subfamily did not differ significantly from the expected 1:1 segregation ratio (Table 2). However, three SV did not fit the expected segregation in 1 (from 3), 2 (from 5) and 2 (from 5) polymorphic subpopulations, respectively, but did fit the expected segregation in the remaining subpopulations. To understand the segregation distortions that were observed for several SV, we sequenced the PCR products. Sanger sequencing resulted in clear chromatograms, excluding the possibility of nonspecific primer binding. Particularly for allotetraploid *B. napus*, it is well known that segregation distortion in certain genome regions can occur within the process of DH production (Pilet et al. 2001; Zhao et al. 2005), either due to linkage with loci impacting DH regeneration or due to homoeologous exchanges in the F1 parent. Linked SNP markers showed the same patterns of segregation distortion, which additionally confirms a biological cause and excludes the possibility of technical issues leading to the observed distortion. However, the 60 DH lines of the subpopulations were produced from up to five single F1 plants. Thus, it is possible that the segregation distortion could be due to de novo SV in individual F1 plants. The SV calls were included in GWAS and allelic effects were calculated (Table 3). For SV4, we detected a significant association with flowering time in the multiparental population in all five locations. Effects varied between 0.38 and 1.42 days earlier onset of flowering for genotypes carrying the deletion. Interestingly, a significant effect of the SV1 deletion on flowering time (0.84–1.62 days earlier flowering) was detected in only three out of the five locations (Hadmersleben, Hovedissen and Soerup), whereas the deletion allele of SV5 showed a significant effect (0.52–1.91 days earlier flowering) only in the other two locations (Leutewitz and Nienstaedt).

**Table 2:** Segregation ratios of six SV events coinciding with five flowering-time candidate genes within the investigated QTL regions of the multiparental population (*n* = 354)

SV type		SV size [in bp]	Lorenz x					
			Adriana	Alpaga	JN	DK Cabernet	Galileo	King 10
SV1	Deletion	373	m	m	29:31 (0.796)	m	m	m
SV2	Deletion	64	32:28 (0.606)	m	5:54 (0.000)***	28:32 (0.606)	36:18 (0.014)*	29:31 (0.796)
SV3	Deletion	1361	m	m	31:29 (0.796)	m	m	m
SV4	Deletion	288	43:17 (0.001)***	m	31:29 (0.796)	m	29:25 (0.586)	m
SV5	Deletion	1313	m	m	m	m	34:20 (0.057)	34:26 (0.302)
SV6	Deletion	35	29:31 (0.796)	39:21 (0.020)*	42:17 (0.001)**	30:30 (1.000)	m	28:32 (0.606)

Detailed descriptions of the SV events can be found in Table 3. Numbers in brackets are the probabilities (P*[χ*^*2*^*]*) that the observed data fit the expected 1:1 segregation pattern. Asterisks show level of significance based on χ^2^ test (*p value < 0.05, **p value < 0.01, ***p value < 0.001). m: monomorphic, not tested in this subpopulation

**Table 3:** Allelic effects of the six investigated SV events coinciding with five flowering-time candidate genes from the QTL in the multiparental population (*n* = 354)

SV	SV type	Hadmersleben		Hovedissen		Leutewitz		Nienstaedt		Soerup	
		Effect	*R*^2^	Effect	*R*^2^	Effect	*R*^2^	Effect	*R*^2^	Effect	*R*^2^
1	Deletion	− 0.84	7.44	− 1.62	4.85	ns	–	ns	–	− 1.47	8.20
2	Deletion	ns	–	ns	–	ns	–	ns	–	ns	–
3	Deletion	ns	–	ns	–	ns	–	–	–	ns	–
4	Deletion	− 0.38	3.63	− 1.01	4.06	− 1.42	3.99	− 0.51	3.48	− 0.80	4.84
5	Deletion	ns	–	ns	–	− 1.91	6.25	− 0.52	3.55	ns	–
6	Deletion	ns	–	ns	–	ns	–	ns	–	ns	–

Effect numbers describe the change in number of days to onset of flowering. *R*^2^: phenotypic variation (%) explained by the respective SV. ns: nonsignificant

### A novel 288 bp deletion in *BnaFT.A02* associates with flowering time

**Fig. 4:**
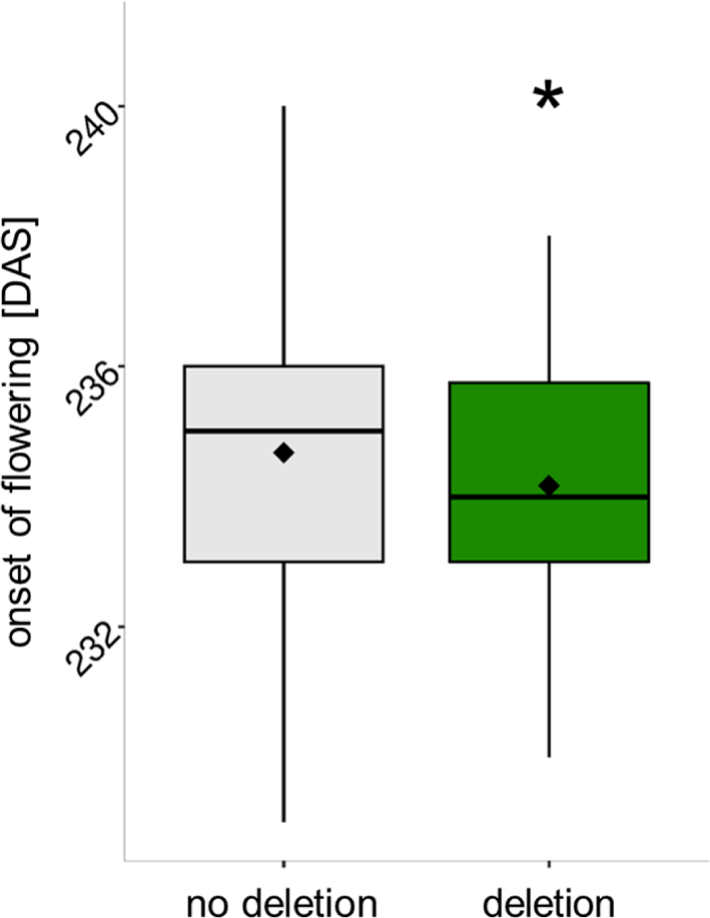
Two boxplots visualize the difference for onset of flowering (days after sowing) depending on the presence/absence of the 288 bp sequence in intron 2 of the gene *BnaFT.A02*. Lines carrying the deletion show an on average earlier onset of flowering of about one day (effect of deletion =  −  0.88) in the *B. napus* multiparental population (*n* = 354). Significant difference is highlighted with green color and asterisks (*p value < 0.05, **p value < 0.01, ***p value < 0.001)

The 288 bp deletion within the second intron of *BnaFT.A02* (SV4) was investigated in further detail because *FT* is a well described key regulator in the flowering-time pathway (Guo et al. 2014; Helliwell et al. 2006; Schiessl et al. 2017c, b; Srikanth et al. 2011) and this specific deletion has not been described before. Validation by Sanger sequencing confirmed that three of the seven parental inbred lines (Adriana, JN, Galileo) carry the 288 bp deletion, whereas four, including the common parent Lorenz, are identical to the reference genome in this gene region. A PCR marker spanning the deletion was used to genotype the entire multiparental population for the SV polymorphism (Supplementary Table S8). Adding this PCR marker as an additional marker to the GWAS revealed a significant association (LOD score = 4.57) explaining 5.64% (*R*^2^) of the phenotypic variation. On average, we observed a slight, but significant earlier onset of flowering (effect of deletion = -−0.88 days) for genotypes having this deletion (Fig. 4). However, at location Leutewitz a stronger effect (− 1.42 days) was observed (Table 3). We screened the 288 bp sequence for transcription factor binding sites using JASPAR (Khan et al. 2018). The analysis revealed binding sites for the *CIRCADIAN CLOCK-ASSOCIATED 1* (*CCA1*) and *LHY* along with several members of the REVEILLE family of transcription factors (Supplementary Table S9), suggesting that this region in the second intron of *BnaFT.A02* may have functional relevance for flowering-time modulation. None of the five other known copies of *BnaFT* in the *B. napus* genome associated to flowering-time QTL, suggesting that they are not responsible for flowering-time variation in this population (Supplementary Table S10).

### Distribution of the 288 bp deletion in genetically diverse winter-type *B. napus*

**Fig. 5:**
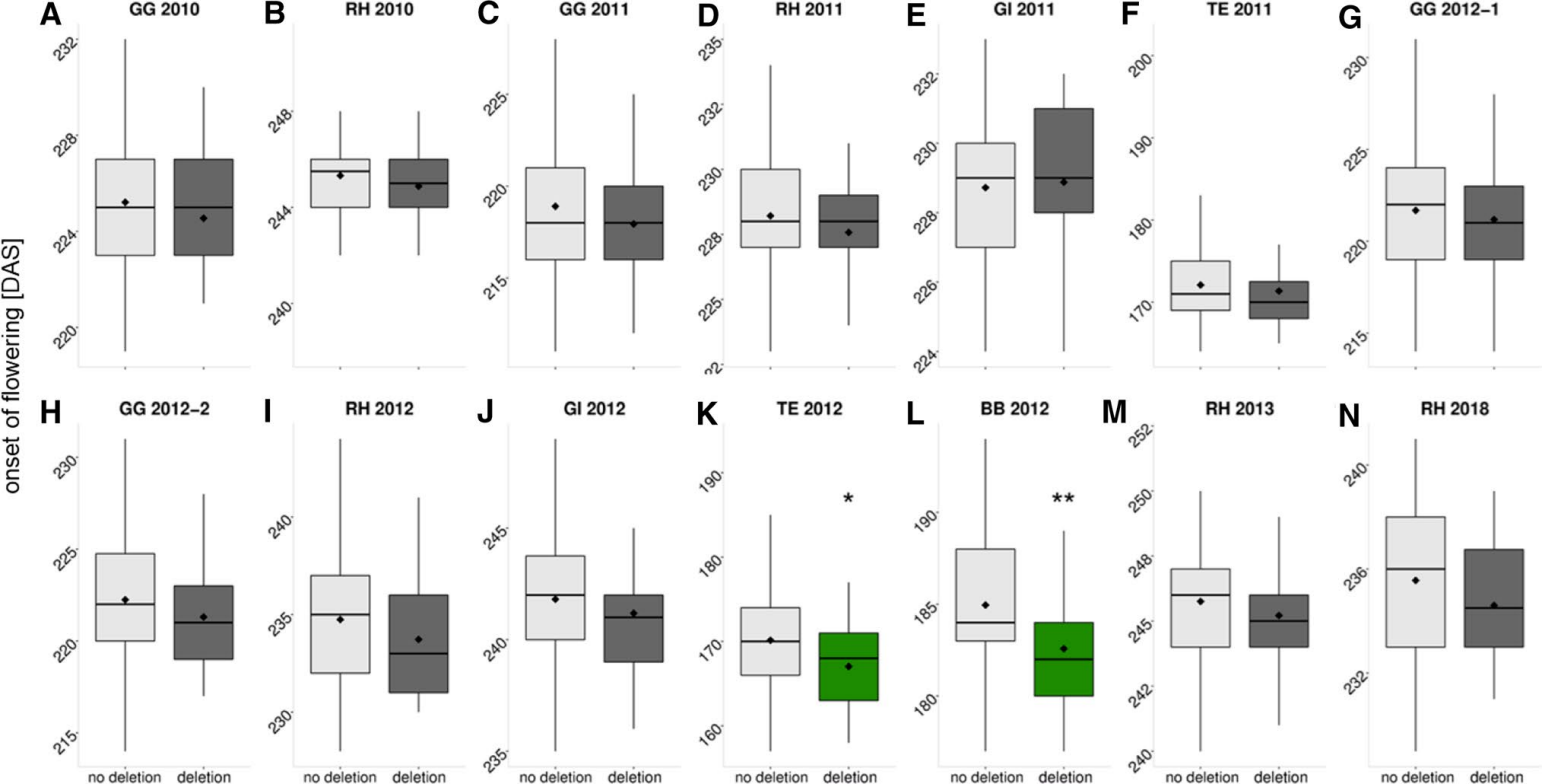
The boxplots visualize the difference in the onset of flowering (days after sowing) depending on the presence/absence of the 288 bp sequence in intron 2 of the gene *BnaFT.A02* in 14 environments for the ERANET-ASSYST diversity set. The environments are abbreviated as follows: Germany: Giessen (GI), Gross Gerau (GG), Rauischholzhausen (RH); Chile: Temuco (TE); China: Beibei, Chongqing (BB), with their respective year of harvest. Black diamonds represent the mean values. Significant differences tested with Student’s *t* test are highlighted with green color and asterisks (*p value < 0.05, **p value < 0.01, ***p value < 0.001) (*n* = 140)

The relevance of the 288 bp deletion in *BnaFT.A02* for flowering-time modulation in winter-type oilseed rape was investigated in a selection of accessions from the ERANET-ASSYST *B. napus* diversity set for which comprehensive flowering-time data were already available (Schiessl et al. 2014, 2015, 2017b). We re-analyzed Illumina short-read data produced by Schiessl et al. (2015, 2017b) from sequence capture of flowering-pathway genes, because the 288 bp deletion was not detectable in the previous analysis, where the Darmor-*bzh* v4.1 reference genome was used for alignment. However, the modified reference sequence without these 288 bp in *BnaFT.A02* allowed us to accurately map the deletion in the diversity set using the short-read sequence capture data (Supplementary Table S11). This approach confirmed the authenticity of the deletion and revealed a widespread distribution in winter-type oilseed *B. napus* cultivars. In addition, the use of short reads enabled us to confirm the borders of the deletion. Of 140 winter accessions used in this study, 34 were found to carry the deletion (NMC < 0.5), whereas 91 were similar to the reference genome (NMC > 1.5). For 15 accessions, analysis of short-read sequencing data coverage and relative copy number calculation did not reveal a clear result (NMC 0.5–1.5). We validated the approach based on short-read sequencing coverage analysis by conducting a PCR on all accessions using the same primers as described above (assay P416, Supplementary Table S3). We confirmed 32 of the 34 NMC deletion calls, whereas two accessions showed contradictory results. Out of 91 accessions without NMC deletion calls, 85 were confirmed. Two showed contradictory patterns, and four accessions were found to be heterozygous. The remaining 15 accessions include three carrying the deletion, eight without the deletion and four which were revealed to be heterozygous. This shows that relative coverage analysis based on Illumina short read data fails to accurately call a 288 bp deletion using 100 bp single-read data. For each of the 14 independent environments, a Student’s *t* test was performed to calculate the phenotypic effect of the deletion assessed using the PCR assay. In this germplasm collection, a significant association of the deletion with flowering time was found only for the locations in China and Chile in 2012, but not for the German locations. However, a trend toward early flowering associated with the deletion was observable in almost all environments (Fig. 5).

## Discussion

Flowering time is an important agronomical trait that has been investigated intensively using QTL analyses and GWAS in oilseed rape. However, most studies on flowering time in *B. napus* investigated germplasm sets with broad genetic diversity including spring, semi-winter and winter-type accessions and focused on major effect QTL. Multiple flowering-time QTL with major effects and high heritabilities have been mapped, and key genes from the flowering and vernalization pathways were repeatedly implicated (Long et al. 2007; Raman et al. 2019; Song et al. 2020; Wu et al. 2019; Xu et al. 2016). In contrast, this study focused on identification of minor QTL which have not already been fixed in adapted elite breeding pools. This was achieved using a multiparental mapping population whose founders were exclusively modern, elite, European winter-type *B. napus* accessions. The aim was to identify small-effect polymorphisms relevant for modulation of flowering time in elite, adapted, winter oilseed rape breeding material. Given this fundamental difference in approach, it is not surprising that our QTL results barely overlap with previous findings. Major differences separating the three major ecotypes (spring, semi-winter and winter types) tend to mask smaller effects responsible for intra-ecotype variation. Some of the significant marker–trait associations for flowering time in the present work were stable only over a limited number of environments, suggesting that QTL effects are minor and may interact with specific environmental factors. Using 158 winter-type accessions, Schiessl et al. (2015) also revealed flowering-time QTL that are highly sensitive to local environments. In that study, none of the central flowering regulators (e.g., *Bna.FT*, *CONSTANS*, *GIGANTEA*) or central vernalization genes (e.g., *Bna.FLC*) was located within QTL regions for the onset of flowering in winter-type *B. napus*. The QTL found by Schiessl et al. (2015) also did not overlap with the QTL we found in our elite winter-type population. This indicates that fine regulation of the onset of flowering in winter-type *B. napus* is environmentally dependent and potentially controlled by multiple different genome regions with small effects.

In contrast to a previous report for disease resistance (Gabur et al. 2018, 2020), inclusion of SNaP markers (array-based presence/absence markers) in the QTL analysis did not result in detection of additional QTL associated with flowering time. This might suggest that SV may not strongly impact flowering-time modulation in elite winter oilseed rape cultivars. However, since we showed associations between SV and flowering-time modulation, it appears more likely that SV events associated with flowering traits are in LD to neighboring SNP markers from the 60 k *Brassica* SNP array in these elite mapping parents. The number of polymorphic SV events we detected among the seven elite parental lines (13,374) is similar to the number of polymorphic SNP detected between these lines using the 60 k *Brassica* SNP array (13,746), suggesting that structural variation is a significant source of sequence polymorphism in elite cultivars with a potentially very important functional role. We found that around 5% of genes are affected by small to mid-scale SV events in each of the seven elite parents, corresponding to the frequency of intragenic SV reported by Chawla et al. (2020) for older winter oilseed rape cultivars. This underlines the importance of functional gene modification during post-polyploidization genome restructuring in *B. napus* (Chawla et al. 2020; Song et al. 2020). The high level of functionally relevant SV polymorphism in modern elite cultivars underlines the high relevance of this kind of variant for selection and breeding and the need to develop a better platform than the 60 k *Brassica* SNP array for population-wide high-throughput genotyping and identification of SV.

Most of the deletions/insertions we identified in candidate genes within flowering-time QTL in elite cultivars reside in introns. With respect to *BnaFT.A02* (*BnaA02g12130D*), one deletion lies in the second intron and another in the putative promoter region. The 1.3 kb deletion in the promoter region of *BnaFT.A02* is associated with the different *B. napus* ecotypes (Chawla et al. 2020), but does not completely explain the differentiation between ecotypes (Schiessl et al. 2019). Because the populations we investigated are exclusively vernalization-dependent, this deletion did not show significant associations with flowering time. In contrast, SV1 (a 373 bp deletion in *BnaA02g00160D*), SV5 (a 1,313 bp deletion in *BnaA02g33650D*) and SV4 (a 288 bp deletion in *BnaFT.A02*) showed significant associations with flowering time. Whereas a direct functional relevance of *BnaFT.A02* is already well established, further functional studies are required to elucidate the potential role of the *B. napus* genes *BnaA02g001160D* and *BnaA02g33650D* in coordination of flowering time. *BnaA02g00160D* is a homolog of *A. thaliana SETDOMAIN GROUP 15* (*SDG15*; *AT5G09790*), a H3K27 methyltransferase which was previously described to be involved in the control of transposal activity and genome stability (Ma et al. 2018). Other methyltransferases have described roles in flowering-time regulation, like *SDG7* (Lee et al. 2015), while *SDG15* was only implicated in pollen formation so far (Raynaud et al. 2006) and is therefore considered less likely to directly influence flowering time here. *BnaA02g33650D* is a copy of *BTB AND TAZ DOMAIN PROTEIN 1* (*BT1*), which is known to plan a role in both male and female gametophyte development (Robert et al. 2009). Hence, this gene is expected to be involved in flower development, but not necessarily in flowering-time regulation, which takes place sometime prior to flower development.

Schiessl et al. (2015) did not detect the QTL harboring *BnaFT.A02* by GWAS using markers from the 60 k *Brassica* SNP array. This might be due to a strong LD decay around SV events or the lower number of accessions and the population structure of the diversity set used in this study. Moreover, the failure to detect a 288 bp deletion using a sequence capture strategy based on short single-read 100 bp Illumina sequencing (Schiessl et al. 2017b) clearly reveals the advantage of long-read sequencing to detect polymorphic small- to mid-size deletions. The 288 bp deletion in *BnaFT.A02* is associated with flowering time at all five field locations in Germany in both the 2017/2018 and 2018/2019 growing seasons. Both years were characterized by extremely warm and dry summers in Germany, in strong contrast to the environmental conditions in the years 2010–2013 during the study of Schiessl et al. (2015). This may suggest a QTL-by-environment interaction that is more active under extreme environments, which in turn might explain why day length is only crucial in the diversity set.

*FT* is one of the key regulators of flowering time in the well-investigated flowering-time pathway (Helliwell et al. 2006; Srikanth et al. 2011). Several studies, particularly in Arabidopsis, elucidated interactions of *FT* with other major genes in this pathway (Faure et al. 2007; Turck et al. 2008). For instance, the single copy of *FT* in Arabidopsis is suppressed by *FLC*, which is downregulated during extended periods of cold temperature to unlock the repression (Helliwell et al. 2006; Searle et al. 2006). However, the allopolyploid *B. napus* genome possesses multiple *FT* orthologs and paralogs with incompletely elucidated function. For example, *BnaFT.A02* is most likely not regulated by *Bna.FLC*. Instead, Wang et al. (2009) showed that the characteristic CArG box to which *FLC* normally binds is missing in this particular *FT* copy, and Guo et al. (2014) demonstrated that *BnaFT.A02* is expressed independently of vernalization. On the other hand, *FT* expression is activated by *CONSTANS* (*CO*), whose activity is promoted by an increase in photoperiod (Kobayashi et al. 1999; Srikanth et al. 2011; Turck et al. 2008; Wigge et al. 2005). In agreement with Schiessl et al. (2015), who demonstrated the day length dependency of *BnaFT.A02*, we found that a deletion polymorphism in *BnaFT.A02* in winter-type *B. napus* only associated with flowering time during trials in Chile and China, where day lengths differed significantly from the German trial locations. This supports a vernalization-independent, day-length-dependent activation of *BnaFT.A02* (Corbesier et al. 2007; Guo et al. 2014; Schiessl et al. 2015).

Expression of *FT* can be detected in leaves and apical meristems (Wigge et al. 2005). Corbesier et al. (2007) proposed the *FT* protein moves from the leaves through the phloem to the meristem. Changes in *FT* expression have been shown to significantly change flowering time in Arabidopsis (Kobayashi et al. 1999) as well as in major crops like barley (Faure et al. 2007), wheat (Yan et al. 2006), maize (Lazakis et al. 2011; Meng et al. 2011) and potato (Navarro et al. 2011). In winter-type *B. napus, Bna.FT* expression changes caused by allelic variants were associated with altered flowering time (Tudor et al. 2020), while high *FT* expression is known to correlate with earlier flowering (Raman et al. 2019). To investigate potential mechanisms by which the 288 bp deletion within the second intron of *BnaFT.A02* might regulate expression of this *FT* ortholog, we searched the sequence spanning the SV for putative regulatory motifs known to be involved in controlling gene expression. Besides the main ‘*FT*-interactors’ *FLC* and *CO*, several other genes are already known to interact with *FT*. For example, *SCHLAFMÜTZE* (Mathieu et al. 2009), *TEMPRANILLO 1* (Castillejo et al. 2008) and *SHORT VEGETATIVE PHASE* (Kobayashi et al. 2007) repress *BnaFT.A02* expression by binding to it. Screening for transcription factor binding sites within the deleted 288 bp sequence revealed binding sites for the well-known circadian clock genes *CCA1* and *LHY*. In addition, *LHY* was found to be located in QTL C03_1. We also noted two unannotated genes with similarity to known flowering-time genes, *GA20-oxidase* (*BnaC02g01710D*) and *CONSTANS-LIKE* (*BnaC02g06280D*), within QTL C02_1 and C02_3, respectively. This suggests that this SV polymorphism may modulate *FT* expression by altering the binding characteristics to one or both of these genes, which are known to act together by the formation of dimers (Lu et al. 2009; Seo et al. 2012) and which have known involvement in photoperiod responses (Fujiwara et al. 2008; Li et al. 2008).

In the past, the prevalence of SV within genes has been mostly ignored in most crop species due to the lack of resolution of short-read resequencing. Recent breakthroughs in long-range sequencing technologies have led to increased application of these tools in recent years, particularly for whole-genome assembly (Michael et al. 2018; Schmidt et al. 2017). To a limited extent, long-read resequencing has also been applied to explore genome-wide SV patterns; however, the focus to date was generally on broad comparisons between small numbers of very diverse accessions (e.g., Chawla et al. 2020; Todesco et al. 2020), or strongly restructured genomes of mapping parents with synthetic background (Gabur et al. 2019). Associations of SV with flowering time, disease resistance and eco-geographical differentiation in diverse oilseed rape *B. napus* accessions (Chawla et al. 2020) revealed a general relevance of SV for breeding. However, the extent, relevance and role of SV for important traits in modern elite cultivars have still not been addressed or documented in detail. Here, we show that SV in key regulator genes of flowering time is associated with flowering-time modulation in elite winter oilseed rape cultivars. The present study stresses the potential value of SV impacting important agronomic traits within a set of well-adapted and strongly selected varieties. Our results suggest that the utilization of markers developed from SV can add additional benefit to breeding programs. Our findings can be used to fine-adjust commercial varieties in terms of flowering time, an increasingly important adaptation trait in the face of climate change.

## Supplementary information

Supplementary file1 (XLSX 96kb)

Supplementary file2 (TIFF 1,61,139kb)

Supplementary file3 (TIFF 1,61,139kb)
